# Rapid Cycle Implementation and Retrospective Evaluation of a SARS-CoV-2 Checklist in Labor and Delivery

**DOI:** 10.1186/s12913-021-06787-5

**Published:** 2021-08-06

**Authors:** Liana Zucco, Nadav Levy, Yunping Li, Toni Golen, Scott A. Shainker, Philip E. Hess, Satya Krishna Ramachandran

**Affiliations:** 1 Department of Anesthesia, Critical Care and Pain Medicine, Beth Israel Deaconess Medical Center, 330 Brookline Avenue, Boston, MA 02215 USA; 2 Department of Obstetrics, Gynecology and Reproductive Medicine, Beth Israel Deaconess Medical Center, Boston, MA USA

**Keywords:** Consolidated framework for implementation research, COVID-19, perioperative checklist, labor and delivery

## Abstract

**Background:**

Preparedness efforts for a COVID-19 outbreak required redesign and implementation of a perioperative workflow for the management of obstetric patients. In this report we describe factors which influenced rapid cycle implementation of a novel comprehensive checklist for the perioperative care of the COVID-19 parturient.

**Methods:**

Within our labour and delivery unit, implementation of a novel checklist for the COVID-19 parturient requiring perioperative care was accomplished through rapid cycling, debriefing and on-site walkthroughs. Post-implementation, consistent use of the checklist was reported for all obstetric COVID-19 perioperative cases (100% workflow checklist utilization). Retrospective analysis of the factors influencing implementation was performed using a group deliberation approach, mapped against the Consolidated Framework for Implementation Research (CFIR).

**Results:**

Analysis of factors influencing implementation using CFIR revealed domains of process implementation and innovation characteristics as overwhelming facilitators for success. Constructs within the outer setting, inner setting, and characteristic of individuals (external pressures, baseline culture, and personal attributes) were perceived to act as early barriers. Constructs such as communication culture and learning climate, shifted in influence over time.

**Conclusion:**

We describe the influential factors of implementing a novel comprehensive obstetric workflow for care of the COVID-19 perioperative parturient during the first surge of the pandemic using the CFIR framework. Early workflow adoption was facilitated primarily by two domains, namely thoughtful innovation design and careful implementation planning in the setting of a long-standing culture of improvement. Factors initially assessed as barriers such as communication, culture and learning climate, transitioned into facilitators once a perceived benefit was experienced by healthcare teams. These results provide important information for the implementation of rapid change during a time of crisis.

**Supplementary Information:**

The online version contains supplementary material available at 10.1186/s12913-021-06787-5.

## Background

### Obstetrics & COVID-19

The Severe Acute Respiratory Syndrome Coronavirus-2 (SARS-CoV-2), which causes the disease COVID-19, was first detected in Massachusetts, USA on 1 February 2020. Statewide spread of the virus was observed in early March and coincided with the declaration of SARS-CoV-2 pandemic by the World Health Organization on 11 March, 2020 [1]. As reports of exponential community transmission became apparent, health care organizations, including hospitals and labor and delivery (L&D) units were prompted to evaluate existing workflow patterns and develop innovations to mitigate risk of viral exposure to patients and staff [2]. The layout and dynamics in L&D units are designed to create a shared experience for family members, while maintaining high levels of readiness for acute deterioration requiring operative delivery. Thus, labor rooms are typically in close proximity to both operating rooms and communal spaces such as the nursing stations and are subject to significant overlapping foot traffic [2]. A single COVID-19 parturient presenting for care at L&D units would pose a considerable risk of viral exposure and spread to other healthcare workers and possibly even other patients, in particular if they required an emergency cesarean delivery.

While clinical guidelines and checklists are core components of patient safety efforts within L&D units [3], the implementation of new guidelines or workflow processes within healthcare is challenging and often hampered by several expected and unexpected barriers [4–6]. The identification of barriers and facilitators is vital in establishing an efficient strategy for change [7], as described in established frameworks such as the Consolidated Framework for Implementation Research (CFIR) [8–10] and Expert Recommendations for Implementing Change (ERIC) [11, 12]. Typically, these analyses are performed prior to the implementation of an innovation. However, formal pre-implementation assessments may not be feasible in crisis situations given the need for urgent implementation of change. Retrospective evaluations of implementation have previously been used to help explain success or failures [13]. The retrospective post-implementation use of CFIR to assess factors influencing implementation outcome has been reported previously [14], but not in the setting of rapid change implementation to manage pandemic spread within hospital units. There is a paucity of literature on factors influencing rapid change implementation during pandemics such as COVID-19, therefore such knowledge may benefit organizations in future planning and preparedness measures.

The aim of our study was to therefore identify the factors that influenced implementation of the perioperative workflow checklist for care of the COVID-19 parturient, by performing a retrospective analysis using CFIR.

## Methods

### i. Design

A qualitative design using CFIR, as the validated assessment tool, was chosen in order to better understand the factors that influenced implementation of a novel workflow. The study was approved by our Institutional Review Board (IRB) at the Beth Israel Deaconess Medical Center. As this was a qualitative study identifying the factors that influenced implementation, and did not constitute human subject research, the requirement for written informed consent was waived. This manuscript conforms to the Standards for Quality Improvement Reporting Excellence (SQUIRE) guidelines and the Template for Intervention Description and Replication (TIDieR) checklist [15, 16].

### ii. Description of Workflow Checklist Implementation (obstetric workflow redesign)

#### Context:

Initial reviews of COVID-19 pandemic preparedness in our hospital identified the need for the redesign of a L&D site-specific perioperative workflow for managing a COVID-19 parturient. Our L&D unit serves as a regional referral center serving an urban, metropolitan area of approximately 4.6 million people, and is the academic teaching hospital for Beth Israel Lahey Health, a state-wide hospital network representing more than 15,000 births annually. As a center for high-risk patients, we anticipated a higher traffic of both diagnosed and suspected COVID-19 patients, in common with earlier experiences at similar units in New York State.

#### Innovation design:

We reviewed the available literature on both SARS-CoV-2 and other related viruses [17], including recommendations on the standards of care from government and professional bodies such as the American College of Obstetrics and Gynecology (ACOG), the Society for Obstetric Anesthesia and Perinatology (SOAP) and the Anesthesia Patient Safety Foundation (APSF) [18–20]. We combined these recommendations with our own organization’s newly designed perioperative workflows for COVID-19 patients to create the L&D workflow for the COVID-19 parturient requiring perioperative care. It was produced as a single page document*,* formatted as a sequential checklist with the intention to be used in real time as a cognitive aid . The checklist was an intentional design decision, documented as an effective means of detailing sequential steps in care [21]; it also fit in with existing local practice of checklist use for pre-operative briefings for all patients going to the operating room on L&D. The intended users of the checklist were staff from nursing, maternal-fetal-medicine, obstetrics, anesthesia and neonatology.

#### Implementation of workflow change:

**Figure 1:**
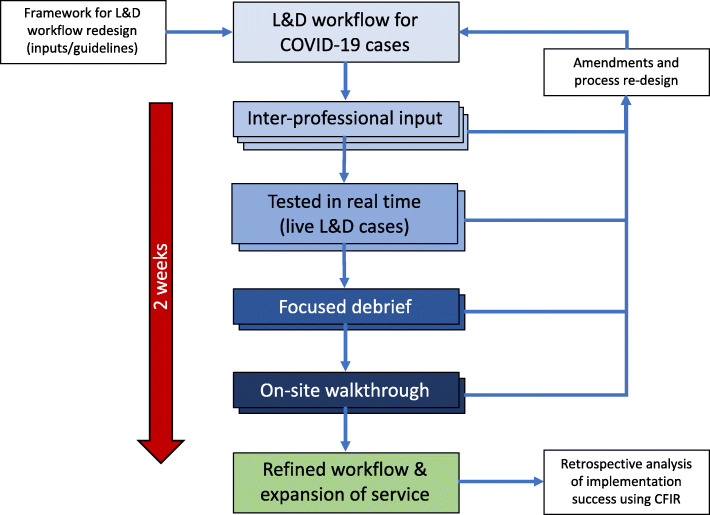
Rapid cycles of change. Schematic representation of rapid-cycle implementation, demonstrating how the individual processes of inter-professional input, testing in real-time, focused debriefing, on-site walkthroughs and iterative re-design contributed to our final refined product. CFIR: Consolidated framework for implementation research, L&D: labor and delivery

Implementation of this innovation took place through a process of rapid cycling over a period of 2 weeks [22–24], described in detail by Li et al, 2020 [2]. The initial workflow draft was disseminated among clinical leaders and stakeholders and underwent one cycle of cognitive redesign. Prior to further refinement, planned testing or wide-scale dissemination amongst providers, its use was urgently requested by clinical leaders to assist in the management of our first live COVID-19 obstetric case. At this time, staff members involved in the case had no formal input into the design of the checklist or training in its use but were coached in real-time to work through the checklist elements. By following the sequence of the checklist, staff were able to safely perform the standard operating procedures, as indicated. Following our first live case, a formal debriefing with all members of the obstetric, anesthesia and perinatal team was conducted using video-conferencing, and specific steps were identified for checklist optimization. Subsequent inter-professional input from the departments of obstetrics, nursing and anesthesia, virtual event debriefings and on-site walkthroughs, several iterations of workflow re-design resulted in our final refined product (Figure 1). Post-case debriefings were performed routinely after-hours and led by the division chiefs of obstetrics, and included staff from nursing, maternal-fetal-medicine, obstetrics, anesthesia, neonatology, and quality and safety. Details on the individual cycles for change are listed (Supplemental Table 1). Our finalized workflow materials are freely available to access online [25].

#### Outcome of implementation:

Adoption of this new workflow was quantitatively defined by documentation of its use during the care of successive COVID-19 parturients over the subsequent weeks, in the medical record. We modified the anesthesia information management system to capture three elements of workflow utilization in a binary (yes/no) fashion: a) patient transport per COVID checklist protocol, b) the intraoperative use of COVID checklist protocol, and c) early postoperative recovery per COVID checklist protocol. Following implementation, we report consistent use of this new workflow for all obstetric COVID-19 perioperative cases; 100% workflow utilization was observed and documented for a total of 23 cases (10 patients who required perioperative care, 13 who required labor analgesia), between March and August 2020. Repeated verbal feedback from frontline clinicians was that the checklist helped with ensuring proper use of PPE, created an environment of safety, and improved coordination and communication among the teams.

### iii. Identification of Factors Influencing Implementation

#### Material:

To identify factors influencing implementation of the redesigned perioperative workflow checklist on L&D, we conducted a detailed retrospective analysis using the CFIR [8, 26, 27]. CFIR classifies operationally defined domains that have been shown to influence implementation success [8], namely, intervention characteristics (e.g.; adaptability, design quality and cost), the outer setting (e.g., external policy, peer pressure), the inner setting (e.g., culture, climate and readiness for implementation), the characteristics of individuals (e.g., knowledge and beliefs about the intervention) and the process of implementation (e.g., planning, engaging, executing and reflecting).

#### Participants:

Our assessment of the implementation experience was mapped against the CFIR constructs and ranked by a panel of 6 experts within our organization. The panel included members of the multidisciplinary team; obstetricians, anesthesiologists and our quality and safety faculty, who are included authors in this study. The first and last authors of this study (LZ and SKR) are not members of the L&D unit.

#### Procedure:

We opted to use a group deliberation approach because of the extensive history of collaborative work that existed in the L&D unit. Given this previous shared knowledge of local context, each construct was evaluated by the group with respect to its likely influence on implementation, and ranked as a facilitator or barrier, having no effect or not applicable to implementation. Virtual group deliberations took place over several days, initially each member of the panel of experts independently reviewed each construct, then as a collaborative discussion facilitated by the senior author and chair of quality and safety division. Disagreements were discussed in two settings, initially through email and then again in person, facilitated by the lead author.

#### Analysis:

In order to compare the relative contribution of each equally weighted construct within each domain at baseline, we transformed these results into a quantitative assessment by allocating a numerical score of 1 to a construct if it acted as a facilitator and 0 if it was considered a barrier or not influencing implementation success. The denominator included all constructs within each domain, apart from those deemed not applicable to the study.

## Results

Evaluation of the implementation experience using CFIR demonstrated the significance of the following domains, when ranked in order of influence as facilitators of implementation success (expressed as a percentage of constructs within each domain): process (89%), innovation characteristics (88%,) inner setting (64%,) characteristic of individuals (40%) and the outer setting (0%). Constructs not applicable to this study included cosmopolitanism, organizational incentives and rewards, and external change agents.

### Facilitators of implementation:

Constructs which **positively** influenced the implementation of this workflow redesign spanned all domains, except the outer setting. The domains of implementation process (Table 1) and innovation characteristics (Table 2) demonstrated the greatest proportion of facilitating constructs. Constructs within the process domain which had a positive influence on implementation included planning and execution, engagement from opinion leaders and key stakeholders, the presence of formally appointed internal implementation leaders and unit champions and repeated reflection and evaluation. Constructs within the domain of innovation characteristics which revealed a positive influence on implementation included the internal trusted source of the innovation, its adaptability, immediate trialability, easy to use checklist design and low cost. Constructs within the inner setting which had a strong influence in facilitating implementation included the structural characteristics of the unit, the implementation climate (tension for change, compatibility, relative priority, goals and feedback) and the readiness for implementation (leadership engagement, available resources) (Table 3).

**Table 1:** CFIR Domain - Process

CFIR Constructs^**a**^	Ranking	Reason for Assigned Ranking	Score
**Planning**	F	The innovation was tested in real-time within the organization, assessed and modified, prior to implementation in L&D. There was a role for all stakeholders in the planning process, tracked the implementation process.	1
**Engagin**g	F	Staff members were engaged with the innovation, invited to use the checklist during live cases, participated in debriefings and did not require repeated attempts to engage. This engagement encouraged feedback and enabled the rapid improvement of steps within the checklist.	1
**Opinion Leaders**	F	Clinical leaders within L&D were engaged with the innovation and were actively involved in each step of implementation, assessment and improvement.	1
**Formally Appointed Internal Implementation Leaders**	F	A formally appointed quality and safety lead (SKR) supported and enabled implementation of this innovation. Clinical leads and local stakeholder buy-in was present.	1
**Champions**.	F	The innovation was informally championed by our surgical obstetric divisional lead.	1
**External Change Agents**	NA	We did not have an outside organization assisting with implementation, this was internally driven and tested.	NA
**Key Stakeholders**	F	Key stakeholders, including a designated quality and safety team, were engaged with the innovation and assisted in the development, implementation, assessment and improvement of the innovation.	1
**Innovation Participants**	NI	The ‘participants’ in this study were considered the patients with confirmed or under investigation for COVID-19. These participants did not impact implementation.	0
**Executing**	F	The redesigned workflow was implemented rapidly, in a concise manner, according to plan.	1
**Reflecting & Evaluating**	F	The implementation team consistently assessed the progress of implementation and the quality of the innovation in order to promote continuous quality improvement.	1

*B* barrier, *CFIR* Consolidated Framework for Implementation Research, *F* facilitator, *NA* not applicable, *NI* no impact.

^a^A description of the CFIR construct is available https://cfirguide.org [28]

**Table 2:** CFIR Domain - Innovation Characteristics

CFIR Constructs^**a**^	Ranking	Reason for Assigned Ranking	Score
**Intervention source**	F	The intervention came from within the organization, it was an internally developed workflow checklist, not from outside policy makers or regulatory bodies.	1
**Evidence, strength & quality**	F	The intervention came from a trusted internal source and from an expert group with awareness of local needs. Though guided by literature from previous epidemics, there was little peer-reviewed evidence of what exactly was needed to promote effectiveness.	1
**Relative advantage**	F	National guidelines and recommendations for managing obstetric COVID-19 patients were collected, synthesised, and disseminated among the stakeholders; however, there was wide variety of interpretation as to the implementation of these in practice. Implementing a sequential checklist was perceived to be faster at producing alignment.	1
**Adaptability**	F	The ability to adapt the innovation to the local obstetric context was clear. Input from multiple disciplines (OB, anesthesia, nursing, NICU) were involved in deciding whether changes were needed to the intervention.	1
**Trialability**	F	Immediate testing was possible. The intervention was used during real cases with the ability to reverse the implementation if required.	1
**Complexity**	B	The workflow was felt to be very complex, involved several aspects of care that were not intuitive and required several iterations to improve performance. It required extra staff members for implementation compared to routine care, which was perceived as a further complication that may have hindered adoption, in particular if staffing levels were low..	0
**Design Quality & Packaging**	F	The initial reception of the innovation was positive and the quality perceived to be high.	1
**Cost**	F	The cost of implementation was the additional manpower needed to ensure the checklist was being followed as the many steps would be impossible to memorise in a short period of time.	1

*B* barrier, *CFIR* Consolidated Framework for Implementation Research, *F* facilitator, *NA* not applicable, *NI* no impact.

^a^A description of the CFIR construct is available https://cfirguide.org [28]

**Table 3:** CFIR Domain - Inner Setting

CFIR Constructs^**a**^	Ranking	Reason for Assigned Ranking	Score
**Structural Characteristics**	F	The intervention took place within the L&D unit, which is a world-leading center of excellence in obstetrics and in anesthesia, and well-established division within the medical center. They have clear processes in place to facilitate quality improvement.	1
**Networks & Communications**	Change over time B to F	Clear lines of communication were not initially evident within the organization regarding this innovation; it was an initial source of frustration for where to locate the most up to date resource. This was rectified over the course of implementation and communicated through the hospital’s COVID intranet. Further communication improvements at the local departmental levels, via intranet, email and teleconferencing permitted inter-professional collaborative work.	0
**Culture**	Change over time B to F	While the culture within the L&D unit was accustomed to the use of checklists, standard operating procedures, and iterative cycle improvement. Additional internal forces along with external pressures of fears and anxiety, were present that affected the cohesion of the unit. Pre-existing egotism and individuality initially impacted implementation negatively. In view of the urgency of COVID-19, recognition that assistance outside of the L&D unit was required, sought and later welcomed over the course of the implementation.	0
**Implementation Climate**	F	Within the organization and within the L&D unit, there was clear receptivity to implementing the innovation. It aligned with existing frameworks already in place, including the use of cognitive aids, checklists, team training and iterative process improvement. Although the checklist and processes were developed quickly, limiting stakeholder buy-in, the implementation climate supported the innovation and valued its use.	1
**Tension for Change**	F	The innovation was absolutely necessary, as the outbreak revealed gaps in our workflow for the COVID-19 parturient.	1
**Compatibility**	F	The innovation was based upon frameworks already used within the organization (e.g.: cognitive aids, checklists) and therefore demonstrated compatibility with organizational values and work processes	1
**Relative Priority**	F	There was clarity in the priority and urgency of this innovation. Given the anticipated surge of potential COVID-19 patients on L&D, implementing this workflow was a priority for all staff.	1
**Organizational Incentives & Rewards**	NA	This innovation was not associated with an external policy or incentive, financial or otherwise.	NA
**Goals and Feedback**	F	This innovation was aligned with organizational and departmental goals, and feedback was obtained to help understand if any gaps existed between the current organizational status and the perceived goal.	1
**Learning Climate**	Change over time B to F	The time pressure resulted, initially, in insufficient time to for reflective thinking and evaluation. Leaders within L&D valued the input of all inter-professional team members and, over time, staff members involved in the implementation felt like a valued partner in the change process.	0
**Readiness for Implementation**	F	The L&D leadership demonstrated a readiness to change; they sought out assistance and innovation.	1
**Leadership Engagement**	F	Organizational leaders demonstrated a dedicated level of engagement and invested adequate time and resource to the innovation. This included the Director of L&D, division director of maternal-fetal-medicine, division direction of OB anesthesia, Anesthesia Executive Vice Chair, and the Vice Chair for quality and safety	1
**Available Resources**	F	Resources, including time, were allocated specifically to the innovation being implemented. Resources in particular: implementation team released from clinical duties to develop and implement this innovation	1
**Access to Knowledge & Information**	B	Access to information regarding the innovation was difficult initially, due to version updates. All information was eventually made readily available throughout the organization through the intranet	0

*B* barrier, *CFIR* Consolidated Framework for Implementation Research, *F* facilitator, *NA* not applicable, *NI* no impact.

^a^A description of the CFIR construct is available https://cfirguide.org [28]

### Barriers to implementation:

Several constructs were felt to **negatively** influence implementation in this study, particularly those from within the outer setting (Table 4). Additional barriers to implementation included the complexity of the change (innovation characteristics, Table 2), baseline culture, climate and communication (inner setting, Table 3) and personal attributes (characteristics of individuals, Table 5).

**Table 4:** CFIR Domain - Outer Setting

CFIR Constructs^**a**^	Ranking	Reason for Assigned Ranking	Score
**Patient Needs & Resources**	Change over time B to F	Despite the purpose of the intervention being focused on managing the patient, it was designed for use amongst the healthcare force. Initially the perceived purpose of the checklist and usefulness for care of the COVID-19 patient was not clear to some staff, creating a barrier for implementation. After the experience gained from a real case and spread of knowledge from the debriefing process after the case, the perceived benefit of the checklist then acted as a facilitator.	0
**Cosmopolitanism**	NA	Networking with external organizations did not apply in this circumstance.	NA
**Peer Pressure**	B	Differences in international and regional guidelines for preparedness and practice for the clinical care of patients with COVID-19 on the L&D Unit resulted in interdepartmental conflicts that impacted behaviours and impacted the readiness for alignment.	0
**External Policy & Incentives**	B	The leadership was in communication colleagues in China, Italy and other centers in the United States. In the early stages the practices and societal recommendations varied considerably, and this affected expectations and prevented shared mental models. This impacted the readiness for alignment.	0

*B* barrier, *CFIR* Consolidated Framework for Implementation Research, *F* facilitator, *NA* not applicable, *NI* no impact.

^a^A description of the CFIR construct is available https://cfirguide.org [28]

**Table 5:** CFIR Domain - Characteristics of Individuals

CFIR Constructs^**a**^	Ranking	Reason for Assigned Ranking	Score
**Knowledge & Beliefs about the Intervention**	NI	Individual stakeholders shared a belief that the intervention was necessary and were seeking an innovation. Obtaining the checklists and processes was challenging initially due to a lack of coordinated communication. There were disagreements with the impact of this construct with equal weighting for facilitator or barrier. It was therefore scored neutrally as having no influence on implementation outcome	0
**Self-efficacy**	F	There was confidence in the ability to implement the intervention and that staff members would be able to use the intervention.	1
**Individual Stage of Change**	NI	Various roles and responsibilities within the organization of staff members affected how they readiness for adoption in the initial stages of implementation.	0
**Individual Identification with Organization**	F	There was broad consensus that all staff members were working toward a common organizational goal.	1
**Other Personal Attributes**	B	Despite several positive traits among stakeholders in terms of willingness to implement changes, expectations toward standard operating procedures and innovation. We identified negative traits such as tribalism, egotism and individualism which affected implementation.	0

*B* barrier, *CFIR* Consolidated Framework for Implementation Research, *F* facilitator, *NA* not applicable, *NI* no impact.

^a^A description of the CFIR construct is available https://cfirguide.org [28]

External pressures created by peer pressure, both locally and internationally, were evident as an early barrier to implementation. Local peer pressure was created by a departmental policy within anesthesia on the appropriate personal protective equipment (PPE). The timing of this change preceded policy change in the L&D unit by a couple of weeks, resulting in general anxiety, disagreement and inconsistencies in inter-departmental guidance that impacted staff behaviors and overall readiness for alignment. Furthermore, external influences from international peer groups, in particular communications from colleagues in China, Italy and other centers across the USA including the Center for Disease Control (CDC), demonstrated a considerable disconnect between the recommendations for care and clinical practice. This affected expectations and resulted in a delay in establishing a shared mental model.

### Constructs which demonstrated a change over time:

Evaluation of our implementation revealed some constructs which demonstrated a temporal change over time, the majority of which were within the inner setting (Table 3). At baseline, constructs such as communication, culture and learning climate initially acted as a barrier to implementation, but then progressed to become facilitators within the space of a few weeks.

With respect to this innovation, clear lines of communication and knowledge of where to access the most up to date information were not evident initially within the organization, which resulted in frustration. This was rectified over the course of implementation and communicated through the hospital’s COVID intranet. Further communication improvements at the local departmental levels, via intranet, email and teleconferencing permitted inter-professional collaborative work.

The group deliberation process revealed that while the culture within the L&D unit was accustomed to the use of checklists, standard operating procedures, and iterative cycle improvement, additional internal forces such as recent staffing changes along with external pressures, fears and anxiety were present that may have influenced the cohesion of the unit. Individual constructs such as "knowledge and beliefs about the intervention" as well as "individual stage of changes" were a source of further deliberation. Stakeholders may initially have shared a belief that an intervention was necessary. However, pre-existing egotism and individualism may have impacted the early learning climate and further impacted implementation negatively. Since both these views provided opposing unweighted impact on the implementation, the arbiters (LZ and SKR) chose to score them as no impact. Yet, we also believe that individual knowledge and beliefs positively influenced sustainability of the workflow implementation, as staff became comfortable with the workflow elements and may indeed have provided a central focus for team behaviors. Additionally, in view of the urgency of COVID-19, assistance outside of the L&D unit was sought and welcomed over the course of the implementation. This openness to external inputs likely influenced individual attitudes as well. Leaders within L&D valued the input of all inter-professional team members during the implementation period. Additionally, through the debrief mechanism, involved staff members felt like a valued partner in the change process. Finally, within the outer setting, the patient’s needs and resources also shifted in influence over time (Table 4).

## Discussion

This paper describes the identification of factors that impacted implementation of a new obstetric workflow checklist, specific for COVID-19 patients in the perioperative setting, through retrospective evaluation using the CFIR established framework.

In our study, constructs within the domains of process implementation and innovation characteristics were overwhelming facilitators of implementation. We believe that transparency in the development and implementation plan along with the design and content of the tool itself were significant influencers. Innovation characteristics found within the tool included the sequential steps of the checklist which ensured that front-line clinicians were able to perform standard operating procedures. Despite the initial impression that our tool was complex, the ability of staff to successfully use the checklist without prior, and extensive, training demonstrates construct validity of the innovation [29]. The immediate and regular testing of our workflow checklist during real COVID-19 obstetric cases by front line staff, enabled us to adapt the tool to meet local requirements [29–31]. In general, cognitive aids should be as concise and clear as possible, and their implementation in other units or environments must include local testing and adaptation for success.

Constructs within the inner setting, such as implementation climate and readiness for implementation, likely acted to support the time pressure. Our institution’s L&D unit is a world-leading center for teamwork and excellence in obstetrics and anesthesia and is a well-established division within the medical center. The unit has a history of clear processes in place to facilitate multidisciplinary quality improvement. A lack of safety culture, policy guideline and senior leadership support have been reported as barriers to implementation during the COVID-19 pandemic [32]. Therefore, we believe that clarity in the prioritization of implementing this innovation along with a readiness demonstrated by senior leaders and stakeholders within our L&D unit and organization facilitated rapid change implementation. A culture of inclusion and teamwork promotes alignment during rapid change, as leaders actively reach out to each other for input [33]. Crisis is a challenging time to develop trust, inclusion and teamwork; a pre-existing culture that includes these characteristics makes it easier to incorporate change, even though staff members within these units may be at different individual stages of change. The effectiveness of implementation in our unit demonstrates that developing a culture of quality improvement, multidisciplinary alignment, and trust has true long-term value. Based on our findings, preparedness measures for rapid change implementation amid a crisis should include an evaluation by organizations of their own inner setting to optimize the learning climate and safety culture.

Implementation in the setting of L&D has generally been previously hampered by individual reluctance to change, pre-existing hierarchical structures and a lack of organizational policy or regulation [34]. Given the context of the pandemic, the influence of some of these usual barriers to implementation may have been lessened. Early publications from international centers and anecdotal reports surrounding COVID-19 left significant room for confusion and interpretation. This may have led to a greater sense of anxiety amongst providers and delays in strategic alignment. This would have typically had a negative impact on implementation, however, in our study a significant observation was that the use of the checklist shortened the preparatory time needed for clinical care, as it provided clarity and unified thinking amongst staff with regards to implementation of clinical recommendations. Thus, the workflow checklist tool was adopted because of the absence of clear global policy. Through this process, it enabled rapid local adoption of the shared mental model and facilitated implementation, despite ongoing conflicting guidance from various national and international sources.

Reflecting on how the pandemic affected implementation outcome, it is worth noting that even in the context of an originally perceived barrier, the urgency and time pressure applied by the threat of an outbreak enabled a rapid transition of impact to facilitate change. The finding of implementation success, despite the presence of perceived barriers within the outer setting, inner setting and amongst the characteristics of individuals, was unexpected. This may be a reflection of the urgency brought about by the COVID-19 pandemic, or perhaps an indication that in the time of crisis, the influence imparted by constructs within these domains are minimal. Furthermore, we noted that drivers for change during the COVID-19 pandemic within our organization are different than during times of routine care. For instance, anxiety around healthcare worker infection rates, the associated stress of rapidly changing local policies and the unknown value of the innovation may negatively impact adherence to guidelines [5]. In contrast, our agile team-centered implementation approach resulted in greater engagement and acceptance of the checklist as a central cohesive factor in enhancing care of these patients.

### Strength and Limitations

In this study we did not perform a comparative pre-post analysis, our evaluation is therefore limited to the post-implementation period. Retrospective evaluations of implementation are often performed to help explain success or failures; they are done at the end of the project and rely on key stakeholder experiences [13, 35, 36]. Rankings of individual constructs was performed through a group deliberation approach, and agreement between raters about the influence of each construct on implementation was consistent, likely reflecting a high level of shared mental model and leadership engagement in the implementation process. We report mainly on the results of qualitative findings, however, in the context of the study objective, qualitative analyses may provide a deeper understanding of the barriers and facilitators for implementation [7]. Quantitative results of implementation are limited to documented or observed use of the innovation but does not reflect a deeper investigation into the precision of its use.

## Conclusions

We describe the factors influencing implementation of a novel comprehensive obstetric workflow for care of the COVID-19 perioperative parturient during the first surge of the pandemic using the CFIR framework. Early workflow adoption was facilitated primarily by two domains, namely thoughtful innovation design and careful implementation planning in the setting of a long-standing culture of improvement. Factors initially assessed as barriers such as communication, culture and learning climate, transitioned into facilitators once a perceived benefit was experienced by healthcare teams. These key factors provide important information for the implementation of rapid change during a time of crisis.

## Supplementary Information

**Additional file 1.**

## Data Availability

Not applicable

## Abbreviations

SARS-CoV-2: Severe Acute Respiratory Syndrome Coronavirus-2
COVID-19: Coronavirus disease 2019
L&D: Labor and delivery
PPE: Personal protective equipment
CFIR: Consolidated Framework for Implementation Research
ERIC: Expert Recommendations for Implementing Change
IRB: Institutional review board
ACOG: American College of Obstetrics and Gynecology
SOAP: Society for Obstetric Anesthesia and Perinatology
APSF: Anesthesia Patient Safety Foundation
CDC: Centers for Disease Control
SQUIRE: Standards for QUality Improvement Reporting Excellence
TIDieR: Template for Intervention Description and Replication
