# Supplementary material for: Rapid Cycle Implementation and Retrospective Evaluation of a SARS-CoV-2 Checklist in Labor and Delivery
Source: BMC Health Serv Res. 2021 Aug 6;21:775. doi: 10.1186/s12913-021-06787-5 (PMC8342983; doi:10.1186/s12913-021-06787-5)
Supplement: Supplementary file 1 — Additional file 1. [file 12913_2021_6787_MOESM1_ESM.docx]

**Supplementary Material**

**Supplemental Table 1: Rapid cycles of change**

| **Cycle 1** |  |
| --- | --- |
| Initial workflow re-design | The initial prototype was created by combining established local protocols for the perioperative management of a COVID-19 patient, existing guidelines for the perioperative management of an obstetric patient, and the existing workflow patterns and logistics within our local center. The focus was on several key aspects of perioperative care that would require modification in the setting of COVID-19; preparation planning and communication, defining new roles, confirming up to date knowledge, logistical considerations. We combined these elements into a single page document that could also function as a cognitive aid or checklist in real time (version 1). The intervention was felt to be a simple, easy to use design and maintained a similar framework to other local checklists in use. We ensured it would be adaptable and required minimal cost. |
| Inter-professional input | Several team members (obstetric, anesthesia, quality) assisted in editing our first prototype. |
| Tested in real-time | Prototype workflow was used in real time, for a live obstetric case that required an emergency Cesarean delivery. It was used by the staff member coordinating the case. |
| Focused debrief | A focused debrief took place immediately post-op in person and virtually (through videoconferencing) within 24hrs. This included all staff members that were involved in the case and invited feedback on the use of the checklist. |
| On-site walkthrough | Workspaces decluttered, emergency equipment bundled into specific carts, appropriate signage on doors. |
| **Cycle 2** |  |
| Workflow re-design | Within 48hrs, a re-designed workflow (version 2) now incorporated elements that were missed or overlooked, the checklist was de-cluttered and unnecessary wording was removed to allow for ease of reading. Other aspects were clarified (e.g.: donning and doffing of personal protective equipment). |
| Inter-professional input | Re-distribution among obstetric and anesthesia teams via email |
| Tested in real-time | The workflow checklist was used during a second live COVID-19 obstetric case. |
| Focused debrief | A focused debrief was performed immediately post-op in person and virtually (through videoconferencing) and inviting all members of the perioperative team involved in the case. |
| On-site walkthrough | Further adjustments to workspaces and processes were noted and included guidance on various logistical details during the case; e.g.: the patient bed and linens. |
| **Cycle 3** |  |
| Workflow re-design | Re-design of the workflow now included a simple algorithm to assist the team in deciding whether or not to treat the patient as a suspected or confirmed COVID-19 patient or whether to proceed with routine care. |
| Inter-professional input | Dissemination of the newly designed workflow took place via email distribution to the department where further feedback was obtained to produce another iteration of the workflow document (version 3) |
| Tested in real-time | The workflow checklist was again used during another live COVID-19 obstetric case. |
| Focused debrief | Continued debriefs took place in person postoperatively, inviting all perioperative team members involved in the case |
| On-site walkthrough | Further walk-through within the obstetric OR revealed deficits in room cleaning and prep |
| **Cycle 4** |  |
| Workflow re-design | Minor edits were again made to the workflow (version 4) in addition to a supplement to describe the specific cleaning protocols for such cases. |
| Inter-professional input | Updated workflows disseminated departmentwide via email and communicated hospital wide through our intranet. Continued input from team members in obstetrics, anesthesiology, nursing and now engineering revealed additional areas for optimization in anticipation of the increase in volume of obstetric cases, and a management plan for emergent gynecological cases. |
| Tested in real-time | Continued use of the workflow checklists in real-time, |
| Focused debrief | Continued regular in person debriefings amongst team members and leadership |
| On-site walkthrough | Development of a COVID-19 sub-unit within L&D, in anticipation of the increase in volume of obstetric cases, and an established management plan for emergent gynecological cases. |
